# Age-Related Reductions in Cerebrovascular Reactivity Using 4D Flow MRI

**DOI:** 10.3389/fnagi.2019.00281

**Published:** 2019-10-17

**Authors:** Kathleen B. Miller, Anna J. Howery, Leonardo A. Rivera-Rivera, Sterling C. Johnson, Howard A. Rowley, Oliver Wieben, Jill N. Barnes

**Affiliations:** ^1^Bruno Balke Biodynamics Laboratory, Department of Kinesiology, University of Wisconsin–Madison, Madison, WI, United States; ^2^Department of Medical Physics, School of Medicine and Public Health, University of Wisconsin–Madison, Madison, WI, United States; ^3^Wisconsin Alzheimer’s Disease Research Center, School of Medicine and Public Health, University of Wisconsin–Madison, Madison, WI, United States; ^4^William S. Middleton Memorial Veterans Hospital, Geriatric Research Education and Clinical Center, Madison, WI, United States

**Keywords:** cerebral blood flow, middle cerebral artery, cerebrovascular conductance, sex differences, neuroimaging

## Abstract

Cerebrovascular reactivity (CVR), is important for determining future risk of cerebrovascular disease. It is unclear if primary aging is associated with reductions in CVR because previous studies often include participants with vascular risk factors. Additionally, the inconsistency in the literature may be due to the inherent difficulty in quantifying intracranial cerebral blood flow and CVR. To address these limitations, we determined the effect of age on CVR in the large intracranial vessels in adults with low vascular risk using state-of-the-art MRI techniques. We also determined if the effect of age on CVR was sex-specific. Young (*n* = 20; 25 ± 3 years) and older (*n* = 19; 61 ± 5 years) healthy, physically active adults participated in the study. CVR was measured in response to hypercapnia using 4D flow MRI, which allows for simultaneous angiographic and quantitative blood flow measurements in the intracranial arteries. Older adults had lower global CVR and CVR in multiple intracranial arteries [right and left internal carotid arteries (ICA), right and left middle cerebral arteries (MCA), and basilar artery (BA)] compared with young adults (*p* < 0.05 for all). In addition, the MCA dilated significantly in response to hypercapnia in young (*p* < 0.05), but not older adults. Young men demonstrated higher global CVR and CVR in multiple intracranial arteries (ICAs, MCAs, and BA) compared with young women and older men (*p* < 0.05 for both); however, CVR did not differ between young women and older women. Our results demonstrate that, using 4D flow MRI, primary aging is associated with lower CVR in adults with low vascular risk. In addition, the effect of age on CVR may be driven by men. The 4D flow MRI technique may provide a promising new alternative to measure cerebrovascular physiology without the limitations of commonly used techniques. Future studies could utilize this MRI technique to examine interventions to maintain CVR with advancing age. This study was registered under clinicaltrials.gov # NCT02840851.

## Introduction

In a healthy brain, increases in the partial pressure of arterial carbon dioxide (CO_2_) rapidly augments cerebral blood flow. The cerebral blood flow response to hypercapnia, termed cerebrovascular reactivity (CVR), is important in determining future risk of cerebrovascular disease (Gupta et al., 2012; Portegies et al., 2014). CVR may also be relevant for vascular cognitive impairment or Alzheimer’s disease (AD) as CVR is lower in patients with AD compared with cognitively normal adults and is progressively worse in AD patients with more pronounced cognitive decline (Shim et al., 2015). Furthermore, reductions in the function of the cerebral blood vessels may appear before the development of other neuropathology (Iadecola, 2004). Because the incidence of cerebrovascular disease and dementia increases with age, it is important to understand physiological changes in CVR associated with primary aging from pathology-related changes in CVR that may result in accelerated decline in brain health.

Despite numerous studies evaluating the effect of aging on CVR in the large intracranial vessels, the topic remains controversial [see Table 3 from the review by Hoiland et al. (2019)]. Many studies rely on whole-brain or single blood artery assessments to measure CVR. In addition, the older participants in previous studies often include those with higher vascular risk (i.e., lack of physical exercise or hypertension), which may confound the effect of age. Furthermore, many studies do not address sex-related differences due to unequal distributions of men and women, and because sex hormones influence the cerebrovasculature, the effect of aging on CVR may be sex-specific (Miller et al., 2013; Deer and Stallone, 2016). Lastly, the controversy in the literature may be due to the fact that the majority of existing work, including our previous work, relies on non-invasive imaging techniques including transcranial Doppler ultrasound (TCD) (Aaslid et al., 1982), which tracks blood velocity of the large intracranial vessels but operates on the assumption that the blood vessel diameter does not change in response to an environmental perturbation such as hypercapnia (Serrador et al., 2000). This idea has recently been challenged by magnetic resonance imaging (MRI) studies indicating that the middle cerebral artery (MCA), which is often used in estimating CVR, dilates during hypercapnia (Brothers and Zhang, 2016; Hoiland and Ainslie, 2016), but the magnitude of dilation may be influenced by age (Coverdale et al., 2017).

Therefore, our objective of this study was to systematically evaluate large intracranial vessel CVR in young and older healthy adults, for the first time, using 4D flow MRI. This is a novel, non-invasive technique that allows for simultaneous angiographic and quantitative blood flow measurements in the intracranial arteries without the use of contrast. 4D flow MRI has been recently validated to study cerebral hemodynamics at rest (Wåhlin et al., 2013; Schrauben et al., 2015; Wu et al., 2016; Clark et al., 2017; Rivera-Rivera et al., 2017; Wen et al., 2019) and during a hypercapnic challenge (Mikhail Kellawan et al., 2016) in humans; however, this approach has not been utilized to address the effect of primary aging. In order to address limitations of previous research, we evaluated CVR using 4D flow MRI in healthy men and women, with low vascular risk, who were physically active and not taking cardiovascular-acting medications. We hypothesized that older adults would have lower CVR compared with young adults when using 4D flow to comprehensively measure cerebral blood flow. In addition, we hypothesized that the effect of aging on CVR would be sex-specific.

## Materials and Methods

### Ethics Statement

All study procedures were approved by the Institutional Review Board of the University of Wisconsin-Madison (2016-0403) and were performed according to the Declaration of Helsinki, including obtaining written informed consent from each participant. This study was registered under clinicaltrials.gov # NCT02840851.

### Participants

Forty healthy adults participated in the study. Participants were recruited via recruitment flyers, including 20 older adults (age range 50–67) and 20 young adults (age range 21–31). All participants had a body mass index (BMI) < 30 kg/m^2^, were non-smoking, normotensive and excluded if they presented with: history or evidence of hepatic, renal, or hematological disease, peripheral vascular disease, stroke or neurovascular disease, cardiovascular disease, diabetes, or other chronic pathologies. In addition, all MRI scans were reviewed by a neuroradiologist (HAR) for incidental findings. Participants were also excluded if they had any contraindication for participating in an MRI study such as an implanted metallic device. Participants were not taking antihypertensive medication or any other vasoactive medications, except for thyroid medication (*n* = 6). Premenopausal women were studied during days 2–6 of their menstrual cycle or during the non-active pill phase of oral contraceptives (*n* = 7). All participants were habitually exercising, such that they met published guidelines for physical activity participation (>150 min/week of moderate intensity aerobic exercise) (Piercy et al., 2018). Exercise participation was determined using a weekly exercise log and a physical activity questionnaire (Godin, 2011).

### Study Procedures

Participants attended a familiarization session on a separate day prior to the MRI visit. During the familiarization session, participants practiced breathing a hypercapnic gas mixture through a facemask. Participants then attended two separate study visits that were conducted in a randomized order. One visit was to measure cardiovascular variables at rest. The other visit was an MRI scan with CVR testing.

Before each study visit, participants arrived after fasting for 4 h, were without caffeine, exercise, and alcohol for 24 h prior and had abstained from non-steroidal anti-inflammatory drugs (NSAIDs) for 5 days. Additionally, participants did not take any over-the-counter medications, vitamins or supplements on the day of the study visits.

### Cardiovascular Measurements

Laboratory tests were conducted in a controlled ambient temperature between 22 and 24°C. Upon arrival, height and weight were measured using a standard scale. After 10 min of supine rest, baseline mean arterial pressure (MAP) was taken in triplicate with a non-invasive brachial blood pressure cuff in the supine position (Datex Ohmeda, GE Healthcare, Fairfield, CT, United States).

To determine vascular health, carotid–femoral pulse wave velocity (PWV) and aortic augmentation index (AIx) measurements were completed utilizing arterial tonometry. High-fidelity pressure waveforms were recorded for at least 10 heart beats non-invasively using a pencil-type Millar Micro-tip pressure transducer from the radial, femoral and carotid arteries (Sphygmocor, AtcorMedical, Sydney, NSW, Australia). An average of 3–5 trials of each PWV and AIx were obtained in succession. Tonometry transit distance from the carotid pulse site, the supra-sternal notch, and the femoral pulse site was measured with a tape measure. PWV was calculated using the intersection tangent foot-to-foot algorithm. An aortic pressure waveform was derived from the radial pulse using the application of a generalized transfer function in order to measure AIx. AIx was then corrected at a heart rate (HR) of 75 beats per minute.

Carotid ultrasound was performed using an 11 L probe with a transmission frequency of 4.5–12 MHz on a GE LOGIQ S8 machine (GE Healthcare, Waukesha, WI, United States). The left carotid artery was imaged in the longitudinal plane in B-mode 1–2 cm below the bifurcation. The carotid intima-media thickness (cIMT) was measured using a semi-automated tracking software offline (Carotid Analyzer for Research, Medical Imaging Applications, Coralville, IA, United States). The average of the far wall IMT over approximately six cardiac cycles is reported. All carotid imaging and analysis was done by the same observer (KBM).

### MR Imaging

MR imaging was performed with a 3T clinical MRI scanner (MR750, GE Healthcare, Waukesha, WI, United States) at the Wisconsin Institutes for Medical Research in Madison, WI. Participants were supine and imaged with a 32-channel head coil (Nova Medical Head Coil, Nova Medical, Wilmington, MA, United States) with a gradient strength of 50 mT/m, and a gradient slew rate of 200 mT/m/ms. Throughout the MRI session, HR and oxygen saturation (SPO_2_) were acquired continuously using a pulse oximeter, and end-tidal CO_2_ (ETCO_2_) was acquired continuously (breath-by-breath) using a nasal cannula. The pulse oximeter and nasal cannula were connected to an MRI compatible monitor (Medrad Veris MR Vital Signs Patient Monitor, Bayer Healthcare, Whippany, NJ, United States).

To determine brain volumes, a T1-weighted structural brain volume (BRAVO) scan was acquired with the following scan parameters: fast spoiled gradient echo sequence, inversion time = 450 ms, repetition time (TR) = 8.1 ms, echo time (TE) = 3.2 ms, flip angle = 12°, acquisition matrix = 256 × 256, field of view (FOV) = 256 mm, slice thickness = 1.0 mm, and scan time ∼8 min.

4D flow MRI data were acquired using a 3D radially undersampled sequence to provide high spatial and temporal resolution (Gu et al., 2005; Johnson et al., 2008). No contrast agent was administered. The scan parameters were as follows: velocity encoding (Venc) = 80 cm/s, FOV = 220 mm, acquired isotropic spatial resolution = 0.7 mm × 0.7 mm × 0.7 mm, TR = 7.8 ms, TE = 2.7 ms, flip angle = 8°, bandwidth = 83.3 kHz, 14,000 projection angles and scan time ∼7 min. The imaging volume covered the right and left MCA, right and left internal carotid arteries (ICA), and basilar artery (BA). Data were acquired continuously, such that a HR of 60 beats/min would provide a sampling of approximately 128 times per heartbeat. Time-resolved velocity and magnitude data were reconstructed offline by retrospectively gating into 20 cardiac phases using temporal interpolation (Liu et al., 2006).

### CVR Protocol

Participants were fitted with an MRI compatible mask that covered their nose and mouth with a one-way valve to prevent re-breathing (Hans Rudolph, Inc., Shawnee, KS, United States). Participants breathed normocapnic air for approximately 10 min during baseline scans. Following normocapnia, participants breathed two stepwise elevations of 4 and 6% inspired CO_2_ administered with oxygen maintained at 21% and balanced nitrogen. MRI acquisition was started after participants’ ETCO_2_ had reached steady state (approximately 2 min) such that each level of CO_2_ was inhaled for approximately 9 min total. This protocol was selected so that MRI acquisition could be completed while ETCO_2_ levels were at steady state. MAP was evaluated every 2 min with an oscillometric non-invasive brachial blood pressure cuff on the left arm (Medrad Veris MR Vital Signs Patient Monitor, Bayer Healthcare, Whippany, NJ, United States).

### Data Analysis

**FIGURE 1:**
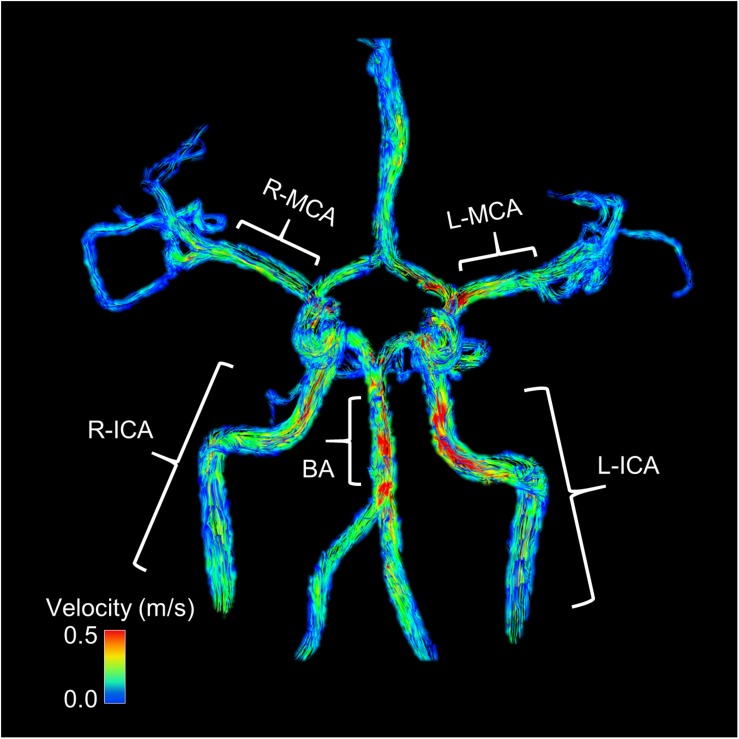
This image shows an example participant’s 4D flow MRI scan during normocapnia. Warmer colors indicate higher flow. Blood flow and vessel cross sectional area (CSA) were averaged along the length of the vessel. The middle cerebral arteries (MCA) were measured along the M1 segment. The internal carotid arteries (ICA) were measured along the cervical and petrous portions below the carotid siphon. The basilar artery (BA) was measured below the superior cerebellar artery and above the bifurcation of the vertebral arteries. Global flow was calculated as the sum of the right and left ICA and the BA flows. End points or branches were removed from the vessel analysis.

Cardiorespiratory variables (ETCO_2_, HR, SPO_2_, and MAP) were averaged over the length of each scan (normocapnia, 4% CO_2_ and 6% CO_2_). Using the T1-weighted scans, brain volumes were segmented in Statistical Parametric Mapping version 12 (SMP12) into gray matter (GM), white matter (WM), and cerebral spinal fluid (CSF). Brain volume was calculated as the sum of GM volume and WM volume. For the 4D flow MRI scans, automatic phase unwrapping was performed in all data sets to minimize potential for velocity aliasing (Loecher et al., 2016). Eddy current correction was also applied as previously described (Schrauben et al., 2015). A time average segmentation mask was generated using both phase and magnitude information. This was done for each subject and for each scan separately (normocapnia, 4% CO_2_ and 6% CO_2_) (Schrauben et al., 2015). Background phase offset corrections were performed in Matlab (The Mathworks, Natick, MA, United States). Individual vessel segmentation of the right and left MCA, right and left ICA, and BA were performed in Matlab using an in-house tool as previously described for semi-automated cerebrovascular flow analysis (Schrauben et al., 2015; Mikhail Kellawan et al., 2017). All vessel segmentation was processed retrospectively. Figure 1 displays an example 4D flow MRI scan of a participant and where each vessel was measured. Blood flow was averaged along the length of each vessel. End points or branches were removed from the vessel analysis. The MCAs were measured along the M1 segment. The ICAs were measured along the cervical and petrous portions below the carotid siphon. The BA was measured below the superior cerebellar artery and above the bifurcation of the vertebral arteries. Global flow was calculated as the sum of the right and left ICAs and the BA. To account for changes in perfusion pressure that may affect blood flow during hypercapnia, cerebrovascular conductance (CVC) was calculated as blood flow/MAP x 100. CVR was quantified as the linear relationship between CVC and ETCO_2_ during hypercapnia in each vessel individually (MCAs, ICAs, and BA) and globally (Barnes et al., 2012; Miller et al., 2018). We also corrected global flow for brain volume [global flow/(GM volume + WM volume)] and used the corrected global flow to calculate CVR. Right and left MCA cross sectional area (CSA) was averaged across the M1 segment of the vessel. All analysis was conducted blind to the subject group (young or older, men or women) and experimental condition (normocapnia, 4% CO_2_ or 6% CO_2_).

### Statistical Analysis

Statistical testing was completed using Sigma Plot for Windows version 13.0 (Systat Software, San Jose, CA, United States). For all variables, normality was assessed using the Shapiro–Wilk test and equal variance was assessed using the Brown–Forsythe test prior to analysis. Participant demographics and baseline characteristics were compared between young and older adults using a one-way ANOVA. A two-way repeated measures ANOVA compared the cardiorespiratory variables between the two groups of interest (young and older adults) during each stage of the hypercapnic protocol (normocapnia, 4% CO_2_ and 6% CO_2_) followed by the Holm-Sidak method to test pairwise comparisons. For our primary analysis comparing CVR between young and older adults, we used a one-way ANOVA. For our secondary analysis, we were interested in determining if any age-related differences in CVR were sex-specific. CVR measures were compared by age group (young or old) and sex (male or female) by a two-way ANOVA followed by the Holm-Sidak method to test pairwise comparisons. Due to the recent data suggesting that the MCA changes CSA during hypercapnia, we also evaluated the changes in the MCA CSA during normocapnia to 6% CO_2_ using a paired *t*-test (two-tailed) in each group of interest (young adults and older adults). We also determined if the change in MCA CSA was sex-specific, using an unpaired *t*-test (two-tailed) between young men compared with young women and older men compared with older women. Statistical significance was set *a priori* at *p* < 0.05.

## Results

### Participants

Participant characteristics are displayed in Table 1. One older woman was excluded from the analysis due to motion artifact on the MRI scans; therefore, data from 20 young adults and 19 older adults are reported. Young and older adults had similar height, weight, BMI, HR at rest, systolic blood pressure at rest and MAP at rest. Older adults had lower brain volume compared with young adults. Older adults, despite having low vascular risk, demonstrated elevated diastolic blood pressure, AIx, PWV, and cIMT compared with young adults. By design, young and older adults were not different in regard to physical activity participation (physical activity questionnaire and calculated MET minutes per week) (Table 1).

**TABLE 1:** Characteristics of Participants.

**Variable**	**Young adults *N* = 20**	**Older adults *N* = 19**	***p*-value**
Female subjects	*N* = 10	*N* = 9	
Age (years)	25 ± 3	61 ± 5	< 0.001
Height (cm)	173 ± 8	172 ± 9	0.68
Weight (kg)	71 ± 10	70 ± 15	0.84
Body Mass Index (kg/m^2^)	23 ± 2	23 ± 3	0.90
Heart rate at rest (beats per minute)	53 ± 8	55 ± 7	0.49
GODIN Questionnaire Score	62 ± 31	61 ± 17	0.95
MET minutes per week	3259 ± 1838	3973 ± 2383	0.30
Systolic blood pressure (mmHg)	120 ± 10	121 ± 11	0.71
Diastolic blood pressure (mmHg)	69 ± 6	74 ± 8	0.03
Mean arterial pressure (mmHg)	86 ± 7	90 ± 9	0.12
Carotid-femoral PWV (m/s)	6.2 ± 1.0	7.8 ± 1.8	< 0.001
AIx (%)	−2.4 ± 9.6	16.1 ± 9.6	< 0.001
cIMT (mm)	0.50 ± 0.08	0.71 ± 0.10	< 0.001
Brain volume (l)	1.20 ± 0.12	1.10 ± 0.13	0.01

*MET, metabolic equivalent, PWV, pulse wave velocity, AIx, aortic augmentation index, cIMT, carotid intima-media thickness. Demographic data are mean ± standard deviation. For PWV and AIx measurements, *n* = 37. Aortic augmentation index was corrected for a heart rate of 75 beats per minute.*

### Physiological Variables

Although ETCO_2_ increased during the hypercapnic stimulus, ETCO_2_ was not different between young and older adults at any stage and the magnitude of increase was similar between groups (Table 2). HR and SPO_2_ were also not different between groups (Table 2). Despite similar MAP at baseline, during the MRI scans, MAP was greater in older adults compared with young adults for normocapnia, 4% CO_2_ and 6% CO_2_ (Table 2) and significantly increased during hypercapnia in older adults.

**TABLE 2:** Physiological variables during hypercapnia.

**Variable**	**Young adults *N* = 20**	**Older adults *N* = 19**	***p*-value**
**Heart rate (beats per minute)**			
Normocapnia	53 ± 1	54 ± 2	0.62
4% CO_2_	56 ± 2^∗^	55 ± 1	0.89
6% CO_2_	58 ± 1^∗^	57 ± 1^∗^	0.65
**End-tidal CO_2_ (mmHg)**			
Normocapnia	40 ± 1	40 ± 1	0.74
4% CO_2_	47 ± 1^∗^	46 ± 1^∗^	0.38
6% CO_2_	49 ± 1^∗^	48 ± 1^∗^	0.25
**Oxygen saturation (%)**			
Normocapnia	98 ± 1	97 ± 1	0.06
4% CO_2_	98 ± 1	98 ± 1	0.46
6% CO_2_	98 ± 1	98 ± 1	0.95
**Mean arterial pressure (mmHg)**			
Normocapnia	94 ± 1	101 ± 2	0.01
4% CO_2_	93 ± 1	104 ± 3^∗^	< 0.001
6% CO_2_	96 ± 1	108 ± 3^∗^	< 0.001

*Data are mean ± standard error. The reported *p*-value denotes group differences at each protocol stage. ^∗^*p* < 0.05 vs. normocapnia within each group.*

### Cerebrovascular Reactivity

**FIGURE 2:**
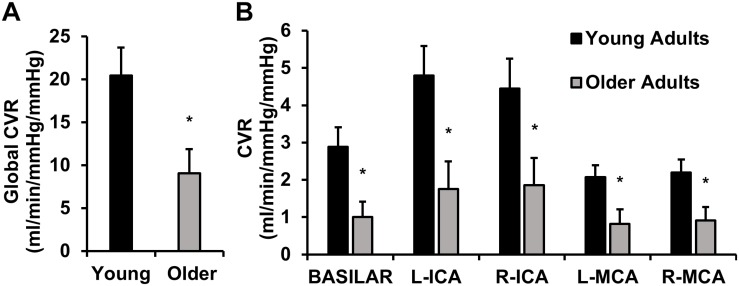
This image displays cerebrovascular reactivity (CVR) to hypercapnia in young and older adults. **(A)** Global CVR in young and older adults. **(B)** CVR in each intracranial vessel of interest [basilar artery, left (L) and right (R) internal carotid arteries (ICA), and L and R middle cerebral arteries (MCA)]. CVR was calculated as the linear relationship between cerebrovascular conductance (CVC) (flow/mean arterial pressure × 100) and end-tidal carbon dioxide during 4 and 6% CO_2_ inhalation. Young adults are shown in black and older adults are shown in gray. Data are mean ± standard error. ^∗^*p* < 0.05. Global CVR was significantly lower in older adults compared with young adults. These results persisted in each vessel of interest (basilar artery, ICAs, and MCAs).

Cerebrovascular reactivity in young and older adults is displayed in Figure 2. Global CVR was lower in older adults compared with young adults (Figure 2A). A *post hoc* observed power calculation demonstrated that the observed power was 80%. These results persisted after correction for brain volumes (*p* = 0.01). Similarly, CVR was lower in older adults in individual intracranial vessels (right and left MCAs, ICAs, and BA) (Figure 2B).

**FIGURE 3:**
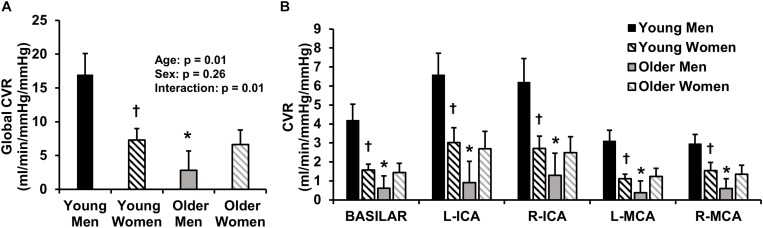
This image displays global cerebrovascular reactivity (CVR) to hypercapnia in young and older men and women. **(A)** Global CVR in young and older men and women. **(B)** CVR in each intracranial vessel of interest [basilar artery, left (L) and right (R) internal carotid arteries (ICA), and L and R middle cerebral arteries (MCA)]. CVR was calculated as the linear relationship between cerebrovascular conductance (CVC) (flow/mean arterial pressure × 100) and end-tidal carbon dioxide during 4 and 6% CO_2_ inhalation. Young men are shown in black, young women are shown in black stripes, older men are shown in gray and older women are shown in gray stripes. Data are mean ± standard error. ^∗^*p* < 0.05 compared with young of same sex. †*p* < 0.05 compared with men of same age. Young men demonstrated higher global CVR than younger women and older men. No sex-differences in global CVR were present in older adults. These results persisted in each vessel of interest (basilar artery, ICAs and MCAs).

We also sought to determine if age-related differences in CVR were sex-specific. Figure 3 displays the global CVR of young and older men and women. Young men demonstrated higher global CVR compared with older men (Figure 3A). There were no differences in global CVR between young women and older women. Thus, the age-related differences in CVR were only present in men. Young men also demonstrated greater CVR compared with young women (Figure 3A); however, there were no sex-differences in CVR in older adults. A *post hoc* observed power calculation demonstrated that the observed power of the interaction (age and sex) was 70%. In addition, young men demonstrated higher CVR compared with young women and compared with older men in each individual intracranial vessel (right and left MCAs, ICAs, and BA) (Figure 3B).

### MCA Cross Sectional Area

**FIGURE 4:**
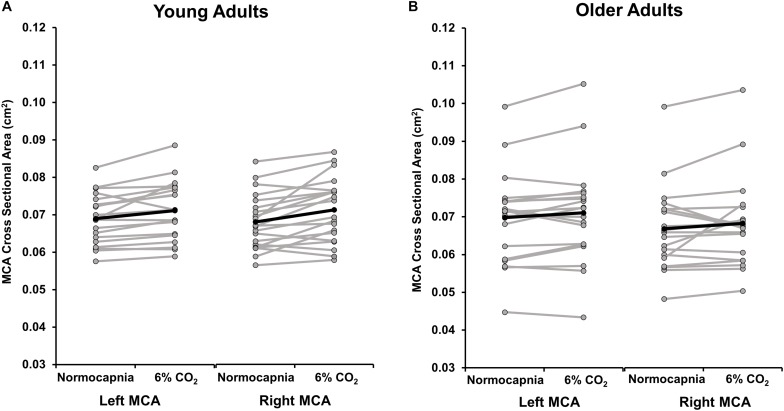
This image displays the cross sectional area (CSA) of the left and right middle cerebral artery (MCA) during normocapnia and hypercapnia (6% CO_2_ inhalation) in **(A)** young and **(B)** older adults. The group average response is shown in black. Gray lines demonstrate individual responses. On average, the left MCA CSA significantly increased from normocapnia to 6% CO_2_ in young adults (*p* = 0.004) but the change in CSA was not significant in older adults (*p* = 0.08). The right MCA CSA significantly increased from normocapnia to 6% CO_2_ in young adults (*p* = 0.002) but the change in CSA was not significant in older adults (*p* = 0.19). There were no differences in the response to hypercapnia between the right MCA CSA compared with the left MCA CSA in young (*p* = 0.28) and older (*p* = 0.72) adults.

Because of the emerging evidence suggesting that the MCA may dilate during hypercapnia (Verbree et al., 2014; Brothers and Zhang, 2016; Hoiland and Ainslie, 2016; Coverdale et al., 2017; Al-Khazraji et al., 2019), we quantified the CSA of the MCA during normocapnia and 6% CO_2_. Group means and individual data are shown in Figure 4. There were no differences between the right MCA CSA response and the left MCA CSA response to hypercapnia in young (*p* = 0.28) or older (*p* = 0.72) adults. In young adults, the CSA of the averaged right and left MCA increased from normocapnia to 6% CO_2_, demonstrating significant MCA vasodilation (normocapnia 0.069 ± 0.001 cm^2^; 6% CO_2_ 0.072 ± 0.002 cm^2^; *p* = 0.001). A *post hoc* power calculation demonstrated that the observed power was 96%. Yet, in older adults the CSA of the averaged right and left MCA did not change significantly between normocapnia and 6% CO_2_ (normocapnia 0.068 ± 0.002 cm^2^; 6% CO_2_ 0.070 ± 0.003 cm^2^; *p* = 0.09). A *post hoc* power calculation revealed that the observed power was 40%.

Because of the sex differences in CVR, we performed a subsequent analysis to determine if the MCA CSA response to CO_2_ was sex-specific. The average CSA showed significant MCA dilation from normocapnia to 6% CO_2_ in young men (normocapnia 0.073 ± 0.002 cm^2^; 6% CO_2_ 0.076 ± 0.002 cm^2^; *p* = 0.02) and young women (normocapnia 0.065 ± 0.002 cm^2^; 6% CO_2_ 0.067 ± 0.002 cm^2^; *p* = 0.02); however, there was no difference in the magnitude of CSA change between groups (*p* = 0.28). The change in MCA CSA in response to hypercapnia was not significant in older men (normocapnia 0.074 ± 0.003 cm^2^; 6% CO_2_ 0.076 ± 0.004 cm^2^; *p* = 0.13) or older women (normocapnia 0.062 ± 0.002 cm^2^; 6% CO_2_ 0.063 ± 0.003 cm^2^; *p* = 0.48) and the magnitude of CSA change was not different between groups (*p* = 0.47).

## Discussion

This is the first study to utilize state-of-the-art 4D flow MRI to comprehensively determine CVR in young and older healthy adults. Our most salient finding is that CVR was lower in older adults compared with young adults, when measuring macrovascular cerebral blood flow responses and accounting for perfusion pressure. The impaired CVR response in older adults was consistent in the right and left ICA, right and left MCA, BA, and globally. Our secondary finding was that the age-related differences in CVR were only apparent in men, and not women. Furthermore, sex-differences exist in CVR in young adults, such that young men had greater CVR compared with young women. Our third finding is that the MCA, which is commonly evaluated in CVR studies, dilated significantly in young adults, but not older adults, in response to hypercapnia, though this analysis had low power. Collectively, our results demonstrate that primary aging is associated with lower cerebral vasodilatory function and suggest that simultaneous angiographic, blood flow, and arterial blood pressure measures to quantify cerebrovascular responses may be necessary to appropriately investigate age-related differences in the cerebral circulation.

### CVR and Aging

Although many studies have reported an age-related reduction in CVR, others have reported similar or even increased CVR (summarized in this review by Hoiland et al., 2019). The existing literature regarding cerebrovascular function and aging is fraught with methodological limitations including: (1) the study population (e.g., confounding vascular risk factors, medication use, unequal distribution of men and women, and physical activity levels); (2) limiting the assessment to one artery; or (3) failure to consider the hypercapnia-induced changes in arterial blood pressure. The novelty of the present study is that we utilized state-of-the-art techniques to address these experimental and technical limitations, including simultaneous angiographic and blood flow measurements of multiple intracranial arteries, as well as determining the diameter of the MCA during hypercapnia. Our goal was to isolate the effects of primary aging, and we did this by including participants who were healthy and habitually physically active without underlying disease or cognitive complaints. We also included an approximately equal distribution of men and women. The older adults in our study were in the middle-aged range (average age of 61 years). It is likely an older population would demonstrate larger age-associated differences in CVR compared with a group of young adults. Utilizing 4D flow MRI, older adults demonstrated lower CVR to hypercapnia, and less dilation of the MCA, compared with young adults, indicating impaired cerebrovascular function. Impaired cerebrovascular function with primary aging has important implications for the etiology of cerebrovascular diseases, including stroke and AD, as reductions in CVR could precede neurological pathology (Iadecola, 2004; Kisler et al., 2017; Toth et al., 2017).

In addition, we included vascular health measurements of AIx, cIMT, and carotid-femoral PWV. Indeed, healthy older adults demonstrated higher central arterial stiffness and greater cIMT compared with young adults. It should be noted that, despite these age-associated differences, the values in older adults are considerably lower than many other reports in the literature (Niiranen et al., 2017) indicating the overall health of the older adults included in this study. Importantly, it has been suggested that stiffening of the central vessels could be a mechanism of primary aging resulting in decreased CVR. Central vessels are responsible for dampening the pulsatile forces that occur with each cardiac contraction and when the vessels stiffen, excess pulsatile energy could be transmitted into the cerebral microcirculation leading to microvascular damage (Mitchell et al., 2011; Watson et al., 2011; Cermakova et al., 2019). Future studies should further explore the influence of central arterial stiffness on CVR, especially in conditions where central arterial stiffness is augmented.

In our study, older adults demonstrated a greater increase in MAP during hypercapnia, despite similar MAP values during rest. This suggests an increased reliance on perfusion pressure to increase cerebral blood flow during a vasoactive stimulus and is consistent with other recent studies (Coverdale et al., 2017; Miller et al., 2018; Tomoto et al., 2019). The compensatory response for augmenting MAP during hypercapnia could be due to a more pronounced sympathoexcitation contributing to the higher arterial blood pressure response. In addition, older adults may have a reduced CO_2_ threshold (García-Río et al., 2007) or rely on increased perfusion pressure in attempt to compensate for reduced cerebral microvessel function (Barnes et al., 2012) and reduced dilation of the cerebral arteries. Ultimately, the mechanism for the age-related reliance on MAP during hypercapnia and age-related reduction in cerebrovascular function are unknown and warrant further investigation so that effects of primary aging can be distinguished from pathological processes.

### Sex-Differences in CVR

Because men and women exhibit distinctive features of cerebrovascular disease etiology and clinical outcomes (Carter et al., 2012; Haast et al., 2012), and sex hormones influence the cerebrovasculature (Deer and Stallone, 2016), we sought to determine if age-related differences in CVR were sex-specific. Our findings were twofold. First, age-related differences in CVR were present in men but not in women. Second, young men demonstrated higher CVR than young women. This suggests that there may be sex-differences in CVR in young adults and that the effect of age on CVR could be driven by men.

We reported higher CVR in young men compared with young women. These findings are similar to previous observations using TCD in our laboratory (Barnes, 2017) and in another study using blood-oxygen-level-dependent (BOLD) MRI (Kassner et al., 2010). However, they are discrepant with several previous TCD studies (Karnik et al., 1996; Kastrup et al., 1997, 1999; Oláh et al., 2000; Peltonen et al., 2015; Madureira et al., 2017). Importantly, these studies vary in experimental technique (i.e., acetazolamide administration vs. CO_2_ inhalation), menstrual cycle control, and do not have an assessment of MCA CSA, which could vary by sex. In our study, the average MCA CSA change did not vary by sex, with both young men and women demonstrating significant MCA vasodilation to 6% CO_2_; however, this was in a relatively small sample and should be repeated with high resolution MRI. A possible explanation for sex-differences in CVR in young adults may be due to differences in vasodilatory reserve. Because previous studies have shown that baseline perfusion in young women is greater than men (Liu et al., 2012, 2016; Ghisleni et al., 2015) and that resistance is low, young women may have a lower sensitivity to a stimulus. Thus, they may require a greater stimulus to initiate vasodilation of the cerebral microvasculature compared with men. In addition, the mechanisms for increasing cerebral blood flow during the vasodilatory stimulus may differ by sex. For example, in a small sample of post-menopausal women, cyclooxygenase inhibition had a greater effect on cerebral vasodilatory capacity compared with age-matched men, suggesting a greater reliance on vasodilatory prostaglandins (Miller et al., 2013). Also, post-menopausal women with higher vascular risk (a history of preeclampsia) demonstrated reduced CVR with parity-matched controls (Barnes et al., 2018), but sex-specific conditions are often not considered in cerebral blood flow regulation studies. Furthermore, chemoreceptor sensitivity and the subsequent sympathetic nervous system response to hypercapnia may be sex-specific (Usselman et al., 2015). As this study was not designed to provide mechanistic insight into why sex differences in CVR are apparent, our interpretations are speculative. Future studies are necessary to examine mechanistic differences in cerebral blood flow regulation in relation to sex-specific etiology of cerebrovascular disease.

### MCA Cross Sectional Area During Hypercapnia

Many studies (Matteis et al., 1998; Schwertfeger et al., 2006; Galvin et al., 2010; Zhu et al., 2013; Oudegeest-Sander et al., 2014; Tomoto et al., 2019), including our previous work (Barnes et al., 2012; Miller et al., 2018), utilized TCD to quantify the MCA velocity response to hypercapnia, which relies on the assumption that the MCA does not change in diameter during hypercapnia. This assumption has been recently challenged (Verbree et al., 2014; Coverdale et al., 2017; Al-Khazraji et al., 2019) and was a *Journal of Physiology* CrossTalk debate (Brothers and Zhang, 2016; Hoiland and Ainslie, 2016). Therefore, the CSA of the MCA during hypercapnia is important to consider and makes TCD and MRI studies difficult to compare.

Interestingly, the increase in CSA of both the right and left MCA during hypercapnia was only apparent in young adults (∼3% increase in the CSA of the left MCA and ∼5% increase in the CSA in the right MCA). We reported no significant changes in CSA of the MCA in older adults, and the magnitude of change in CSA in response to hypercapnia was more variable in older adults, though this analysis had low power. These changes in CSA were modest but consistent with studies utilizing different neuroimaging techniques to address this issue (Verbree et al., 2014; Coverdale et al., 2017; Al-Khazraji et al., 2019). Collectively, this work suggests that vasodilation to hypercapnia within the intracranial vessels may be age-dependent.

If the MCA is dilating during hypercapnia, which our study suggests, it is likely that CVR in young adults is frequently under-estimated when MCA velocity, not flow, is quantified using TCD. The inability to accurately quantify blood flow would contribute to the discrepancies in the literature and highlights the need for other techniques to address the effect of age on CVR and to examine how interventions can improve CVR. Importantly, the 4D flow MRI technique does not require the time-consuming and user-dependent need for manual placement of measurement planes prior to scanning. It also allows for an average CSA change across the entire vessel. In addition, it does not require manual delineation of the vessel diameters. This is especially important in aging individuals when the vascular smooth muscle cells undergo structural changes (Monk and George, 2015). In summary, though TCD has the potential to evaluate brain blood velocity in a variety of settings without the limitations of an MRI scanner, studies evaluating CVR in the intracranial vessels should consider that CVR could be under-estimated if vessel diameter is not quantified. Future studies should explore the potential age-related changes in CSA of the intracranial vessels during hypercapnia or other potentially vasoactive stimuli.

### Limitations

This study had several limitations. In order to compare our findings to previous TCD studies, make certain that participants were at steady-state during MRI acquisition, and ensure participants could tolerate the CO_2_ stimulus for a longer duration than most studies (up to 9 min) we used concentrations of 4 and 6% CO_2_. These concentrations likely did not evoke maximal dilation. Future studies could address age-related differences as well as sex differences using different vasodilatory stimuli. In addition, our findings regarding sex-differences in CVR (observed power, 70%) and hypercapnia induced change in MCA CSA in older adults (observed power, 40%) had low power. These data can inform future studies but should be repeated with higher subject numbers in order to be well powered. Some of the young women in our study were taking oral contraceptives. To date, there is no evidence that oral contraceptives affect CVR using TCD techniques, but that does not exclude the possibility that oral contraceptives affect the ability of the intracranial vessels to dilate in response to hypercapnia. We also included participants taking thyroid medication, which is common in post-menopausal women. It is possible that these medications may be a source of additional variability. In addition, using 4D flow MRI to determine vessel CSA has the advantage of measuring the CSA across the entire vessel without having to manually delineate the lumen; however, since the CSA is determined by the functional outer boundaries of the blood flow velocity profiles, heterogeneous blood flow responses, or partial volume effects could impact the CSA measurements. This methodology has been validated using 2D phase contrast using phantom experiments for given flow rates (Schrauben et al., 2015); however, it is important that future experiments evaluating intracranial vessel dilation also utilize high-resolution MRI (7T), include both black-bold and bright-bold sequences, and validate this technique using changing flow conditions. While we observed that older adults demonstrated an increase in blood pressure response during hypercapnia, we cannot determine if this is due to increased sympathetic activity as we did not measure muscle sympathetic nerve activity, or noradrenaline spill-over during the MRI scan. Although the sample size was small, subject numbers were consistent with previous studies evaluating age-related changes in cerebrovascular function [see Table 3 from the recent review by Hoiland et al. (2019)].

The limitations of the current study were balanced by the experimental strengths. We utilized a novel, state-of-the-art technique (4D flow MRI) to comprehensively evaluate CVR to hypercapnia in young and older healthy adults for the first time. This enabled simultaneous acquisition of flow and CSA data in multiple intracranial vessels at two different levels of hypercapnia (4 and 6%). In addition, we attempted to address limitations of previous research by including participants who were physically active, had low vascular risk, and were free from underlying disease. We also included young women and studied them during the same phase of their menstrual cycle (low-hormone phase). Lastly, we systematically evaluated CVR globally as well as in the major intracranial vessels, including the right and left ICAs, the right and left MCAs, and BA.

## Conclusion

When measured using 4D flow MRI, healthy older adults have lower CVR to hypercapnia and augmented MAP during hypercapnia, compared with young adults. These results suggest that cerebral vasodilatory function is reduced with primary aging. In addition, when young and older adults were analyzed by sex, young men had greater CVR compared with young women, and the age-related differences in CVR were only apparent in men. There were no sex-differences in CVR in older adults. These findings suggest there may be sex-differences in cerebral blood flow regulation. Follow-up studies could utilize this technique to examine interventions to improve CVR. In addition, future research could evaluate how age-related reductions in CVR may contribute to the pathophysiology of cerebrovascular diseases including AD and dementia.

## Data Availability Statement

Because of the large size of the data and the required expertise to analyze the data, the raw data supporting the conclusions of this manuscript will be made available by the authors to any qualified researcher who makes reasonable and scientifically feasible requests. The sharing of the specialized, non-standard data, will occur through direct transfer after appropriate institutional data use agreements have been completed.

## Ethics Statement

The studies involving human participants were reviewed and approved by the Health Sciences Institutional Review Board at the University of Wisconsin-Madison. The participants provided their written informed consent to participate in this study.

## Author Contributions

KM, OW, and JB conceived and designed the research, and interpreted the results of the experiments. KM, AH, LR-R, and JB performed the experiments. KM, LR-R, SJ, HR, and JB analyzed the data. KM prepared the figures. KM and JB drafted the manuscript. All authors edited and revised the manuscript, approved the final version of the manuscript, and agreed to be accountable for all aspects of the work.

## Conflict of Interest

The authors declare that the research was conducted in the absence of any commercial or financial relationships that could be construed as a potential conflict of interest.
